# Physicochemical Attributes Related to Sensory Characteristics of Long-Term Aged Korean Traditional Soy Sauce (Ganjang)

**DOI:** 10.3390/foods13203326

**Published:** 2024-10-20

**Authors:** Yang Soo Byeon, Jungmin Oh, Kyung-Hyung Ku, Mi Jeong Kim, Sang Sook Kim

**Affiliations:** 1 Research Group of Food Processing, Korea Food Research Institute, Wanju-gun 55365, Republic of Korea; b.yangsoo@kfri.re.kr; 2 Enterprise Solution Research Center, Korea Food Research Institute, Wanju-gun 55365, Republic of Korea; jmoh@kfri.re.kr (J.O.); khku@kfri.re.kr (K.-H.K.); 3 Department of Food and Nutrition, Changwon National University, Changwon 51140, Republic of Korea; 4 Interdisciplinary Program in Senior Human Ecology, Changwon National University, Changwon 51140, Republic of Korea

**Keywords:** long-term aged ganjang, descriptive attributes, volatile compounds, consumer acceptability, partial least square regression correlation

## Abstract

This study investigated the physicochemical properties influencing the sensory characteristics of long-term aged ganjang. Eight ganjang samples aged 3, 10, and 15 years were obtained from three different manufacturers and analyzed for physicochemical characteristics, sensory profiles, and consumer acceptability. The proximate composition (moisture, ash, protein, and crude fat), total solids, salinity, acidity, pH, color (L, a, b, chrome, and hue), 27 free amino acids (FAAs), and volatile compounds were analyzed. Quantitative descriptive analysis was performed by 11 trained panelists for sensory profiles and 102 consumers evaluated consumer acceptability (overall, appearance, odor, taste/flavor, and mouthfeel). The results demonstrated a positive correlation between the aging period and increases in total solids, proteins, crude fat, acidity, color hue, FAA, major volatile compounds, and overall consumer acceptability. Specifically, correlation maps by partial least squares regression between descriptive attributes and FAAs or volatile compounds revealed that these components significantly affected consumer acceptability. Furthermore, sensory attributes such as color intensity, viscosity, sweetness, umami, and aftertaste were positively correlated with consumer preference, whereas attributes such as greenish-brown color, fish sauce-like flavor, and moldy notes were negatively correlated. Overall, these findings could be utilized to enhance the marketability and consumer appeal of long-term aged ganjang products by providing objective information supporting premium values.

## 1. Introduction

Ganjang, a traditional fermented soy sauce, is a soybean product widely produced and consumed in various Asian countries [1]. Increasing consumer interest in health and wellness has considerably increased the consumption of ganjang, including ganjang. This could be ascribed to the numerous health benefits associated with ganjang. Studies have shown that ganjang can help prevent type 2 diabetes, improve serum lipid levels, and reduce the risk of cancer, inflammation, oxidative stress, weight, and blood pressure [2,3,4,5,6,7]. These health benefits are largely attributed to the bioactive compounds produced during the fermentation process [8]. The fermentation of ganjang increases the isoflavone content in aglycones and generates several other beneficial bioactive compounds, thereby enhancing its positive health effects [2,9].

The fermentation process not only produces beneficial bioactive compounds, but also contributes to complex flavor and mouthfeel profiles distinguished by its salty, subtly sweet, viscous, and deeply savory attributes [10,11,12]. Ganjang has played a crucial role in these properties for centuries by enhancing the flavor of a wide variety of dishes [11]. Unlike soy sauce products from other countries, traditional Korean soy sauce, ganjang is made from meju, a fermented soybean block containing wild microorganisms, along with salt and water, and relies entirely on natural fermentation without the addition of other starters [13]. The quality of fermented soy products, such as ganjang, may be influenced by various factors, including raw materials, manufacturing region, aging period, and fermentation conditions. These factors can substantially affect the taste, flavor, and functional components of the final product [8,14]. Among these factors, the Maillard reaction, which occurs during the aging of ganjang, generates a bitter taste and aromatic components, adding to the complexity of the flavor profile and contributing to consumer preference [15].

Previous studies have primarily focused on profiling microbial communities and metabolites in ganjang [16]. Studies have also examined consumer preferences and sensory attributes of different types of ganjang [11]. However, information on how long-term aging affects ganjang quality and consumer preferences is limited. Understanding how these factors change over extended aging periods is crucial, considering that the premium value of long-term aged ganjang products is widely accepted without scientific evidence.

This study aimed to investigate the impact of long-term aging on the quality and consumer preferences for ganjang. By examining the sensory attributes, physicochemical properties (including proximate composition, color, salinity, pH, and acidity), amino acid composition, and volatile compounds that develop during the aging process, this study seeks to provide a comprehensive understanding of how aging affects ganjang.

Overall, long-term aged ganjang is highly valued despite the limited information available. This study provides objective insights into the value of long-term aged ganjang by examining its sensory characteristics and physicochemical properties that influence consumer acceptability.

## 2. Materials and Methods

### 2.1. Ganjang Samples

Table 1 shows the eight ganjang samples used in this investigation, which were manufactured by three certified traditional food producers. Of the 36 sample manufacturers examined in a previous study conducted by Byeon et al. [11], three were selected based on their possession of long-term aged (10–15 years) ganjang samples. All eight ganjang samples were made solely of meju and were aged in porcelain pots for 3, 10, or 15 years using traditional methods. All ganjang samples used in this study have been awarded the Certification of Quality of Traditional Foods (CQT) in Korea [11].

### 2.2. Proximate Analysis, Color, pH, Acidity, and Salinity

Proximate analyses were performed on the total solid content (TS), ash, crude fat, and total nitrogen (TN) using the Association of Official Analytical Chemists (AOAC) methods 925.10, 945.28, 991.36, and 920.53 [17], respectively. Protein content was estimated by measuring the total nitrogen (TN) using the Kjeldahl method (AOAC 920.53), and applying the following conversion formula:

Protein (%) = TN (%) × 6.25

where 6.25 is the standard conversion factor for soy products. This factor is based on the assumption that soy proteins contain 16% nitrogen on average. The results were reported as percentages of weight per weight (% *w*/*w*).

The color parameters L*, a*, b*, c*, and h° represent the lightness, redness, yellowness, chroma, and hue, respectively, and were quantified using a CM-5 spectrophotometer (Konica Minolta, Tokyo, Japan). Measurements were taken under standard illuminant D65 and 10° standard observer. The L* value represents lightness (0 = black, 100 = white), a* indicates redness (+) to greenness (−), and b* represents yellowness (+) to blueness (−). Chroma (C*) is calculated as C* = $\sqrt{{a*}^{2}+{b*}^{2}}$, and hue angle (h°) is determined using h° = tan^−1^(b*/a*). The pH was measured utilizing a 720A pH meter (Orion Research, Inc., Boston, MA, USA).

The titrimetric method of AOAC 973.42 was used to measure the total acidity (TA). Total acidity (TA) was measured by titrating the sample with 0.1 M sodium hydroxide until it reached a pH of 8.2. The results are represented as grams of lactic acid equivalents per 100 mL. The Mohr method was used to measure salt content (g/100 mL) as described by Hwang and Kim [18].

### 2.3. Free Amino Acids

Free amino acids (FAAs) in the ganjang samples were analyzed according to the method described by Byeon et al. [11]. A high-speed amino acid analyzer, L-8800 (Hitachi High-Tech Co., Tokyo, Japan), was attached to an ion exchange column #2622SC PF 4.6 mm i.d. × 60 mm (Hitachi High-Tech Co., Tokyo, Japan). The mobile phase consisted of PF1, PF2, PF3, PF4, PF-RG, R-3, C-1, ninhydrin solution, and a buffer solution (Wako Pure Chemical Industries, Osaka, Japan). The samples were extracted in a rotary shaker for 30 min using 100 mL of 16% trichloroacetic acid solution. Subsequently, the mixture was centrifuged at a speed of 740× *g* for 15 min at a temperature of 4 °C. Each supernatant-obtained extract was filtered with a 0.2 μM syringe filter (Life Science, Boston, MA, USA). The filtered samples were injected into the analyzer system using a post-column for ninhydrin derivatization. As an internal standard, a solution mixture was prepared using 1:1 (*v*/*v*) type AN-II #015-14461 and B #016-08641 (Wako Pure Chemical Industries, Richmond, VA, USA) at various concentrations. The content of FAAs was calculated by systematically comparing them with relevant standards using the EZChrom Elite software ver 3.3.2 SP2 (Agilent Technologies, Santa Clara, CA, USA). Measurements were repeated twice and presented as averages.

### 2.4. Volatile Compounds

The volatile compounds in the ganjang samples were measured using gas chromatography–mass spectrometry (GC-MS) with GC-2010 Plus and GCMS-TQ 8030 instruments (Shimazu, Tokyo, Japan). To extract volatile compounds, 1 g of ganjang, 1 mL of distilled water, and an internal standard (methyl-pentanol) were stirred in a 20 mL vial at 40 °C. The mixture was then extracted using solid-phase microextraction (SPME) fibers (57348-U; Supelco, Bellefonte, PA, USA). Following the extraction of the volatile chemicals, the SPME fibers were moved to the GC inlet. A GC system was utilized for the chromatographic separation, employing a DB-WAX column with dimensions of 30 m × 0.25 mm i.d. × 0.25 μm film thickness (J&W Scientific, Santa Clara, CA, USA). The GC column was initially kept at a temperature of 40 °C for a duration of 2 min. Subsequently, the temperature was increased to 230 °C at a rate of 11 °C per min and maintained at that level for an additional 4 min. The temperature of the injector was adjusted to 230 °C, and a flow rate of 1 mL/min was implemented for the helium carrier gas. The MS was operated in Q3 scan mode at a voltage of 0.1 KV, with the ion source and interface temperatures set at 200 and 250 °C, respectively. The volatile compounds found by GC-MS were identified by systematically comparing them with reference mass spectra from the NIST 11 and Wiley 9.0 mass spectral libraries and using retention indices. The concentrations of volatile compounds were semi-quantified using 2-methyl-1-butanol detected in the sample as a reference for quantification. This was performed by comparing the peak area of each compound with that of the known mixes. Measurements were repeated three times and presented as average and standard deviation.

### 2.5. Sensory Analysis

Descriptive analysis and consumer tests were approved (KFRI 2023-04-004) by the institutional review Board of the Korea Food Research Institute. All sensory research studies adhered to the ethical guidelines of the Declaration of Helsinki [19].

#### 2.5.1. Descriptive Sensory Analysis

For the descriptive analysis, 11 panelists (females, aged 37–49 years) were selected from 30 preliminary panelists based on their ability to discriminate and describe tastes and flavors in a screening test. These panelists participated in 15 training sessions (2 h per session, twice a week). The selection of descriptive sensory attributes was based on sensory evaluations conducted with trained panelists. Descriptive sensory attributes were developed during panel training for the ganjang samples used in this study. And final descriptive attributes were determined by agreement among panelists during the panel discussion session. During panel training, 32 descriptive sensory attributes were established for eight ganjang products. The attributes were categorized into appearance (viscosity, precipitates, degree of color, and greenish-brown color); odor (jocheong, sour, savory, doenjang, burnt, spicy, moldy, and fish sauce); taste/flavor (sweetness, sourness, bitterness, saltiness, umami, doenjang, savory, and fish sauce); mouthfeel (thickness, spiciness, and astringency); and after-taste/after-mouth feel (sweet, sour, bitter, salty, umami, savory, tingling, stinging, and astringent). The definitions, reference materials, and strengths of the reference materials for each attribute are presented in Supplementary Table S1. The samples were evaluated in an individual sensory booth equipped with a computerized data collection system (Korea Food Research Institute, Wanju-gun, Republic of Korea) using a 15 cm line scale (0: none to 15: very strong; each end of the scale was anchored). The panelists evaluated four samples in each session and were given 10 min to test each sample, after which they were required to rest for 10 min to prevent sensory fatigue. Ganjang samples (40 g) were provided in a 2 oz transparent cup coded with three-digit random numbers. The samples were covered with a lid and presented monadically and randomly to prevent any bias. Filtered water and cooked rice were used for palate cleaning. Additionally, a 0.2 mL teaspoon and a spit cup were also provided. Three replicates were conducted for the evaluation.

#### 2.5.2. Consumer Test

This study included 102 participants aged 20–65 years. Of them, 41 were male and 61 were female. Before completing the questionnaire, each participant provided informed consent, confirming their willingness to participate in the study and their lack of allergies to soybeans. For the consumer test, individual sensory booths were utilized, and the data were directly input into a computerized data-gathering system (Compusense Inc., Guelph, ON, Canada). As a sample presentation protocol, each ganjang sample (10 g) was presented in a 2 oz transparent cup covered with a lid, and a 0.2 mL teaspoon was provided as previously described in the descriptive sensory analysis. Each sample was evaluated for 10 min, with a five-minute break between samples to prevent fatigue of the assessors and allow the sensory receptors to recover. In addition, filtered water and cooked rice (50 g) were provided as palette cleansers between samples. Four samples were evaluated per session and each test session was conducted over two consecutive days. To eliminate any possible bias, samples were presented in accordance with the Williams Latin Square design [20]. The participants received monetary remuneration. The participants were instructed to assess the acceptability of the ganjang samples by rating each sample on a nine-point hedonic scale for appearance, odor, taste/flavor, mouthfeel, and overall acceptability.

### 2.6. Statistical Analysis

For descriptive attributes, consumer data, physicochemical properties, amino acids, and volatile compounds, an analysis of variance (ANOVA) was performed to determine differences among the ganjang samples. When a difference among samples was found, significant differences were calculated using the Student–Newman–Keuls (SNK) multiple comparison test at *p* < 0.05. First, partial least squares regression (PLS-R) analysis was conducted to determine the physicochemical characteristics (xs) affecting the acceptability (Ys) or sensory attributes of long-term aged ganjang products. Second, PLS-R analysis was conducted to determine the FAAs or volatile compounds (xs) affecting the sensory attributes (Ys) of long-term aged ganjang products. All statistical analyses were performed using Xlstat statistical software ver. 2022.2.1. (AddinSoft, Paris, France).

## 3. Results and Discussion

### 3.1. Proximate Analysis, Color, pH, Acidity, and Salinity

Changes in the proximate composition, color, pH, acidity, and salinity of the eight ganjang samples with different aging periods (3, 10, and 15 years) and manufacturers (A, B, and C) are shown in Table 2. Statistically significant differences (*p* < 0.001) were observed among the eight ganjang samples in terms of moisture, TS, ash, protein, crude fat, color (L*, a*, b*, c*, and h°), salinity, pH, and acidity (Table 2).

The moisture and TS contents of the eight ganjang samples exhibited substantial variation with aging period and manufacturer, ranging from 43.3 (C10) to 72.1% (B03) for moisture and 27.9 (B03) to 56.7% (C10) for TS, implying that as the aging period increased, moisture evaporated, and the TS content increased (Table 2). These results are consistent with the findings of Joo, et al. [21], who observed a decrease in the moisture content and an increase in the pure extract content as the aging duration increased to two years. They also explained that the TS content affects the flavor and taste of ganjang [21].

The protein content ranged from 4.9 (C03) to 13.0% (C15) and showed an increasing trend with age. Although the lipid content of all the ganjang samples was below 1%, it increased over longer aging periods. The protein content of ganjang may be represented by the TN content, which is an important indicator of ganjang [14,15]. Similar to the protein and lipid contents, acidity also increased with longer aging periods, supporting the findings of Zhang, et al. [22], who reported that the TA content of ganjang increased as fermentation progressed, mostly due to the elevated production of organic acids by lactic acid bacteria.

In the results of this study, the color values for L*, a*, and b* ranged from 66.7 (C15) to 69.2 (B15), −0.06 (B15) to 0.52 (B03), and −0.38 (C10) to 0.33 (C15), respectively. Although statistically significant differences were observed, they were not significantly influenced by the aging period or manufacturer. In the case of manufacturers, the color of the ganjang samples produced by manufacturer C (Doguri, Jeju, Republic of Korea) varied more significantly with the aging period than those manufactured by A (Isaacfarm, Gongju, Republic of Korea) or B (Onggojib, Gunsan, Republic of Korea). Magishi, et al. [23] found that Maillard reaction products produced by the aging process could lead to an increase in the a and b values of ganjang. The h° values were determined using the formula (h°) = atan2(b, a), and an increase in the h° value could contribute to the intensity of the brown color in the soy source. In this study, the h° values increased from 30.3 to 79, 26 to 89.4, and 36.5 to 69.0 for manufacturer A, B, and C, respectively. Thus, implying that the h° values increased with the aging period. Salinity and pH values exhibited an overall downward trend with prolonged ganjang aging. During extended aging, microbial activity increases significantly, leading to the enhanced production of organic acids, which consequently results in a lower pH. Additionally, the ongoing breakdown of proteins and other compounds during this process may contribute to a reduction in salinity, as these substances are metabolized by the microbial communities. This phenomenon has been reported in another study, highlighting the complex interactions between microbial dynamics and the physicochemical properties of fermented products, such as ganjang [24].

### 3.2. Free Amino Acids

Free amino acids play a crucial role in the unique taste and aroma of fermented foods such as ganjang [25,26]. Table 3 shows the FAA contents of ganjang with different aging periods and manufacturers, showing significant differences among the ganjang samples. The content of (FAAs in A03 was lower than that in A10, and similar trends were observed in samples of manufacturers B and C, indicating that all analyzed FAAs increased with age, regardless of the manufacturer. These results may be attributed to the activity of proteases released by microbes during the process of making ganjang production, where longer aging periods result in higher FAA production [15].

Overall, the most abundant FAAs in the aged ganjang samples was glutamic acid, followed by lysine, alanine, leucine, valine, proline, aspartic acid, isoleucine, serine, and threonine, consistent with the findings of Byeon et al. [11]. Glutamic acid content was lower in C03 (226.6 mg/100 g) than in A03 (600.9 mg/100 g) and B03 (727.1 mg/100 g). Among samples A10, B10, and C10, A10 had the highest glutamic acid content (1275.9 mg/100 g), whereas C10 (841.1 mg/100 g) had the lowest. A03 had the highest aspartic acid content (318.2 mg/100 g), whereas B03 had the lowest (30.4 mg/100 g). Among the ganjang samples aged for ten years, A10 (649.8 mg/100 g) had the highest aspartic acid content. Notably, B15 (642.5 mg/100 g) had higher aspartic acid content than C15 (325.5 mg/100 g). Among the A10, B10, and C10 ganjang samples, glutamic acid and aspartic acid contribute primarily to the umami taste, providing the most important function as a condiment [27]. Among the samples, A10 and B15 exhibited the highest levels of FAA related to savory taste, whereas the increase in C03 with aging was less pronounced. Zhao et al. [25] reported that threonine, serine, glycine, lysine, and alanine were associated with sweetness. Leucine, valine, and isoleucine are associated with bitterness, as described by Diez-Simon et al. [28]. Another study has reported that peptides containing proline, glycine, alanine, valine, leucine, tyrosine, and phenylalanine contribute to the bitterness of fermented foods [25]. In the present study, C15 had the highest alanine and proline levels, whereas B15 had the highest glycine and serine levels. Leucine, valine, and isoleucine, which are associated with bitterness, were abundant in various samples, contributing to the complexity of the ganjang flavor profile. B15 had the highest levels of arginine, histamine, isoleucine, methionine, phenylalanine, threonine, and valine, while leucine and lysine were highest in C15. Although bitterness can be perceived as unpleasant, it adds depth to the overall taste profile of ganjang [29].

### 3.3. Volatile Compounds

Table 4 presents various volatile compounds identified in the eight ganjang samples, which differ by aging periods and manufacturers. These compounds included 10 alcohols, 7 aldehydes, 10 ketones, 8 pyrazines, 6 hydrocarbons, 6 fatty acids, 5 esters, and 4 compounds related to nitriles, sulfurs, or phenol. As shown in Table 4, the characteristics of volatile compounds vary depending on the aging period, influencing the overall flavor profile of the ganjang. The concentrations of volatile compounds were semi-quantified using the detected 2-methyl-1-butanol as a reference. This approach, where a single compound is used to estimate the concentrations of others, has been employed in studies such as the formation of volatile compounds in rice fermentation and the determination of key compounds in soy sauce fermentation. While different compounds were used, the methodology is comparable.

The most abundant volatile compounds in the ganjang samples were the aldehydes 2-methylbutanal, 3-methylbutanal, and acetic acid. Feng et al. (2015) reported that 2-methylbutanal and 3-methylbutanal exhibited the highest average odor activity values (>100) among 27 commercial ganjang products [30]. In this study, 2-methylbutanal and 3-methylbutanal in samples A10, B15, and C15 were found to be higher than those in samples A03, B03, and C03, respectively, supporting the results reported by Song et al. [31]. They reported that aldehydes such as 2-methylbutanal and 3-methylbutanal increased with prolonged ganjang fermentation [31]. Another study found that 3-methylbutanal has the highest odor activity value in ganjang [26]. Reportedly, 3-methylbutanal imparts a malty aroma that positively influences consumer preferences [32].

Our findings on the prevalence and increase in these aldehydes with aging are consistent with recent comprehensive studies on soy sauce aroma compounds. For instance, Gao, et al. [33] in their review of Chinese soy sauce research, and Gao et al. [34] in their study of selenium-enriched soy sauce, both highlighted the importance of 3-methylbutanal and 2-methylbutanal as key aroma compounds. This consistency validates our approach, despite the limitations of using a single internal standard and not employing GC-Olfactometry (GC-O).

Lee et al. [35] investigated the changes in volatile compounds in ganjang over 12 months of fermentation. They reported that the contents of most compounds, such as acids, aldehydes, benzene and benzene derivatives, esters, lactones, pyrazines, pyrones, and pyrroles, tended to increase, whereas those of alcohols and ketones decreased with the fermentation period [35]. Contrary to the findings of Lee et al. [35], the results of this study showed that alcohols such as 2-methylbutan-1ol and 3-methylbutan-1ol were abundant in ganjang samples from manufacturers A and B. However, they were not found in those from manufacturer C, suggesting that the influence was more related to the manufacturer than to the aging period. Among the alcohols, furan-2-ylmethanol tended to increase with aging, although it was not found in A03 or C03. This phenomenon was also observed in ketones. Butan-2-one, the major ketone in ganjang samples, was higher in A10, B15, and C15 than in A03, B03, and C03, implying an increase in the aging period. Thus, the results of this study do not agree with the report by Lee et al. [35] that ketones decrease during the fermentation period. This may be explained by the different aging periods of the ganjang samples. The aging periods for the ganjang samples in this study were 3, 10, and 15 years, and the aging period was up to 12 months, as reported by Lee et al. [35].

Among the esters, methyl-2 hydrobenzoate was the major ester in the ganjang samples. In the present study, the methyl-2-hydrobenzoate content tended to increase with age, as reported by Lee et al. [35]. Methyl-2 hydrobenzoate in A03, B03, and C03 was lower than that in A10, B15, and C15, respectively. Acetic acid and 3-methylbutanoic acid were detected in all eight ganjang samples. Acetic acids were in the range of 97.6 (B03)–1622.8 mg/mL (C03), while Jang et al. [36] reported a slightly narrow range of volatile acetic acids (211.6–1451.2 mg/mL) in traditional ganjang samples aged for three years, which were produced in six different regions in Korea [36]. The range of 3-methylbutanoic acids for ganjang samples used in this study was 89.4 (A10)–188.8 mg/mL (B15). Among the ganjang samples, C03, C10, and C15 contained more acetic acid than the other samples. Acetic acid content in A10 was higher than that in A03. In addition, B15 had a higher acetic acid content than B03. It seems that ganjang samples with longer aging periods contained more acetic acid. However, this trend was not observed in samples C3 and C15. Jang et al. [36] reported that the concentrations of (methyldisulfanyl)methane, 3-methylbutanenitrile, and (methyltrisulfanyl)methane tend to increase with age. The (methyldisulfanyl), 3-methylbutanenitrile, and (methyltrisulfanyl) methane contents in A10 and B15 were higher than those in A03. and B03, respectively. The (methyldisulfanyl) methane and (methyltrisulfanyl) methane contents in C15 were higher than those in C10. (Disulfanyl) methane is a volatile sulfur-containing compound. These sulfides can significantly influence the odor characteristics of Welsh onions, due to the low thresholds of such odor notes (unique cooked onion, cooked cabbage, and garlic-like odor notes), ranging from 0.16 to 1.2 ppb [37,38]. Lee et al. [38] reported that (methyldisulfanyl)methane in heated Welsh onions decreased with the storage period.

Our study, utilizing GC-MS with a single internal standard, provides valuable insights into the volatile compound profile of ganjang over extended aging periods. While this method effectively identifies and semi-quantifies a wide range of compounds, it has limitations compared to more recent techniques like GC-O and multiple internal standard approaches. The primary constraint is our inability to directly correlate identified compounds with specific sensory attributes. However, the consistency of our findings with more specialized correlation studies between volatile profiling and descriptive analysis suggests that our method captures key volatile components effectively, particularly over extended aging periods.

### 3.4. Sensory Characteristics

To verify the changes in the quality and flavor of ganjang during long-term aging and to assess the contributions of the key odor or taste-active compounds, eight ganjang samples were evaluated for sensory characteristics by trained panelists and consumers.

The ANOVA results revealed that 13 descriptive attributes exhibited significant discrimination among the ganjang samples, as shown in Table 5. Among the descriptive attributes, appearance-related attributes varied greatly depending on the degree of long-term aging (*p* < 0.001). The viscosity and degree of color tended to increase in long-term aged ganjang samples. In contrast, the precipitates and greenish-brown color of the ganjang samples decreased with long-term aging. Significant differences were observed in odor-related attributes, such as jocheong, savory, doenjang, burnt, spicy, and moldy odors, depending on the ganjang sample. Jocheong, savory, burnt, and spicy odors generally increased in the long-term aged samples.

The consumer acceptability of the ganjang samples is shown in Table 6. The overall acceptability of ganjang samples tended to increase with longer aging periods. Odor/aroma, taste/flavor, and mouthfeel also showed similar trends. A03 and A10, produced by Isaacfarm, were less affected by the aging period in terms of consumer acceptability. However, ganjang samples from producer’s B (Onggojib) and C (Doguri) showed an increasing trend in consumer acceptability for samples aged > 10 years. As shown in Table 5 and Table 6, ganjangs with attributes such as viscosity, degree of color, jocheong, burnt, and sweetness tended to be liked more by consumers. Table 6 reveals valuable insights into the relationship between aging time and consumer preference across different manufacturers. The data clearly demonstrate that longer aging periods generally correlate with higher consumer acceptability scores across all sensory attributes (overall, appearance, odor/aroma, taste/flavor, and mouthfeel).

For manufacturer A (Isaacfarm), we observe a slight increase in overall acceptability from 3 years (6.10) to 10 years (6.39) of aging, confirming our earlier observation of minimal impact of aging on this producer’s samples. In contrast, manufacturer B (Onggojib) shows a more pronounced improvement, with overall acceptability increasing from 4.61 at 3 years to 5.76 at 10 years, though slightly decreasing to 5.66 at 15 years. Manufacturer C (Doguri) demonstrates the most significant improvement with aging, rising from 4.66 at 3 years to 6.10 at 10 years, and further to 6.13 at 15 years.

This pattern is consistent across most sensory attributes, with notable improvements in odor/aroma and taste/flavor scores as aging time increases. The data suggest that the optimal aging period may vary between manufacturers, with some reaching peak acceptability at 10 years (e.g., manufacturer A), while others continue to improve up to 15 years (e.g., manufacturer C).

### 3.5. Correlation of Consumer Acceptability and Physicochemical Characteristics or Descriptive Attributes

**Figure 1:**
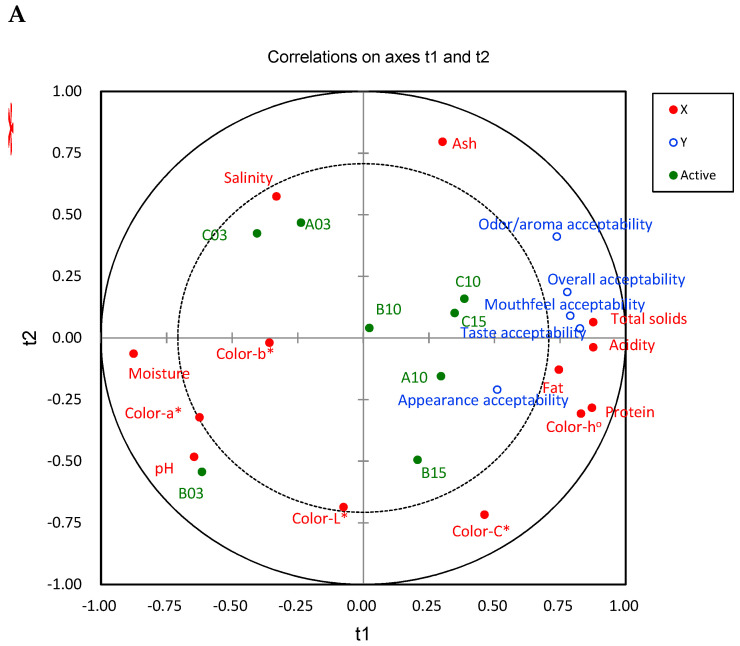

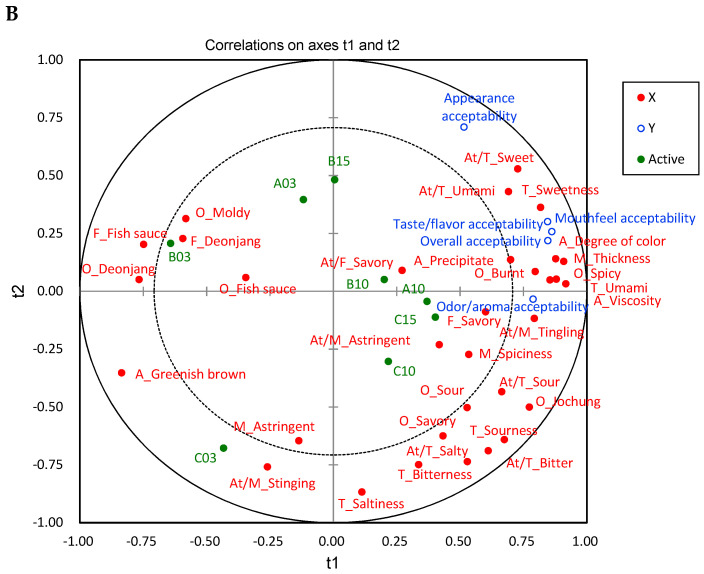
Correlation map by PLS-R with consumer acceptability (Ys) and (**A**) physicochemical characteristics (Xs) or (**B**) descriptive attributes acceptability (Xs) of eight ganjang samples (A_: appearance, O_: odor, F_: flavor, M_: mouthfeel, At_: aftertaste, T_: taste, At/M_: aftertaste mouthfeel). The inner and outer ellipses in the plot represent 50% and 100% of the explained variance, respectively.

The influence of physicochemical properties, such as the proximate composition, pH, acidity, and color of ganjang, on consumer acceptability was determined using PLS-R analysis to identify key properties (Figure 1A). Thirteen physicochemical properties were defined as X variables, whereas five consumer acceptability attributes—overall appearance, odor/aroma, taste/flavor, and mouth feel—were defined as Y variables (Figure 1A). As shown in Figure 1A, the ganjang samples aged for three years (A03, B03, and C03) were positioned on the negative side of dimension 1 (t1), whereas the other samples aged for longer periods were positioned on the positive side of dimension 1 (t1). Compared to the other samples, the A03 and C03 ganjang samples had relatively high salinity, and B03 showed a high correlation with pH, suggesting that consumers tend to dislike ganjang at higher pH and salinity levels. Although prolonged aging of ganjang samples did not exhibit a high correlation with physicochemical properties and consumer acceptability, the levels of total solids, acidity, crude fat, and protein were found to contribute to the overall mouthfeel and taste/flavor acceptability. Based on the consumer acceptability results (Table 6), consumers generally preferred ganjang samples positioned in positive dimension 1 (t1), suggesting that the characteristics highly correlated with physicochemical and consumer acceptability (total solids, acidity, crude fat, and protein, as well as consumer acceptability, such as overall, mouthfeel, and taste/flavor acceptability) could be important quality indicators (Figure 1A).

Figure 1B presents a PLS plot showing the relationship between the descriptive analysis results and consumer acceptability for eight ganjang samples. In this plot, the X-axis represents 13 descriptive attributes, while the Y-axis corresponds to consumer acceptability. For the descriptive attributes studied in Section 3.6 and Section 3.7, we focused on odor, taste, and flavor attributes that have been consistently identified as important in ganjang sensory profiles and those that showed significant variation across our samples. Acceptance attributes such as overall, appearance, odor, taste/flavor, and mouthfeel, are mainly situated on the positive side of t1, suggesting that t1 encapsulates the key elements affecting consumer acceptability. Sensory characteristics like ‘viscosity_A’, ‘degree of color_A’, ‘burnt_O’, spicy_O’, sweetness_T’, ‘umami_T’, ‘thickness_M’, ‘sweet_At’, ‘umami_At’, and ‘tingling_At’ were closely associated with consumer acceptance, indicating their significant influence on how the ganjang samples were received. In contrast, the B03 and C03 samples, which showed the lowest consumer acceptability, were positioned on the negative side of t1. The C03 sample was characterized by low acceptability due to mouthfeel attributes such as ‘astringent_M’ and ‘stinging_At/M’. Similarly, the B03 sample exhibited low consumer liking due to negative odor and flavor attributes, including ‘moldy_O’, ‘doenjang_F’, ‘fish sauce_F’, ‘doenjang_O’, and ‘fish sauce_O’. Both of these samples were aged for three years, whereas the B10, C10, and C15 samples, which were aged for 10 and 15 years, showed increased acceptability. This suggests that as the aging period increases, mouthfeel, negative odor, and flavor attributes tend to transform into characteristics that consumers prefer, such as ‘savory_F’, ‘astringent_At/M’, ‘spiciness_M’, ‘burnt_O’, and ‘precipitate_A’.

### 3.6. Correlation of Taste/Flavor-Contributing Free Amino Acids with Sensory Characteristics

**Figure 2:**
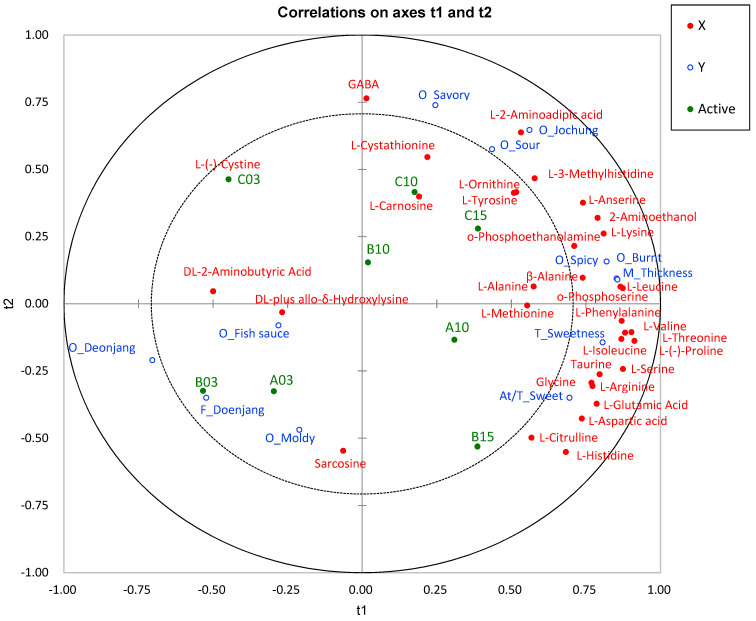
Correlation map by PLS-R with amino acids (Xs) and descriptive characteristics (Ys) of eight ganjang samples (A_: appearance, O_: odor, F_: flavor, M_: mouthfeel, At_: aftertaste, T_: taste, At/M_: aftertaste mouthfeel, M_: mouthfeel). The inner and outer ellipses in the plot represent 50% and 100% of the explained variance, respectively.

PLS-R analysis was conducted to explore the impact of free amino acids that contribute to taste and flavor on sensory characteristics and to identify key components. In this analysis, twenty-seven free amino acids were defined as X variables, whereas 12 descriptive sensory attributes related to taste, flavor, odor, and aftertaste were defined as Y variables (Figure 2). The inner and outer ellipses in the plot represent 50 and 100% explained variance, respectively. Free amino acids (FAAs) positioned between the two ellipses are considered to have a strong association with taste and flavor as sensory attributes [39]. Notably, 22 FAAs and nine sensory attributes were located between the two ellipses, suggesting that they were well explained by the PLS-R model. As shown in Figure 2, samples A10, B15, and C15 were closely grouped together and associated with sensory attributes such as savory, sweetness, and umami in both taste and aftertaste. This finding aligns with that of a study by Baryłko-Pikielna and Kostyra [40], who reported that sweet, umami, and bitter tastes are particularly significant in determining food acceptance or rejection. In particular, ganjang is widely used as a condiment because of its umami and salty taste [41], implying that umami plays a significant role in consumer preferences for ganjang. The PLS-R correlation map in this study further illustrated that glutamic acid and aspartic acid were strongly correlated with sweet and umami aftertastes. Additionally, the findings showed that in aged ganjang, amino acids such as alanine, phenylalanine, serine, isoleucine, leucine, proline, glycine, and *ο*-phosphoethanolamine play a crucial role in imparting umami and sweetness sensory properties, as previously reported by Asanuma et al. [15].

### 3.7. Correlation of Odor-Active Volatile Compounds with Sensory Characteristics

**Figure 3:**
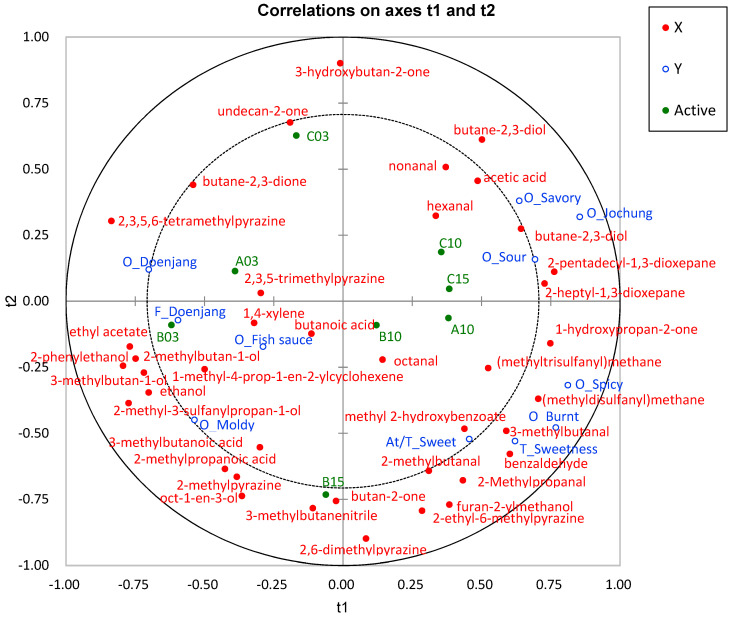
Correlation map by PLS-R with major volatile compounds (Xs) and consumer acceptability characteristics (ys) of eight ganjang samples (A_: appearance, O_: odor, F_: flavor, M_: mouthfeel, At_: aftertaste, T_: taste, At/M_: aftertaste mouthfeel, M_: mouthfeel). The inner and outer ellipses in the plot represent 50% and 100% of the explained variance, respectively.

PLS-R analysis was conducted to investigate the relationship between odor-contributing volatile compounds and descriptive analysis results across various ganjang samples. As shown in Figure 3, the correlation map generated by the PLSR analysis demonstrated that ganjang samples with higher consumer acceptability, such as C10, C15, and A10, were positioned on the positive side of t1. Notably, samples aged more than 10 years tended to shift rightward along the PLS1 dimension, suggesting that prolonged aging significantly influences the volatile compound profile, thereby playing a crucial role in shaping consumer preferences.

Furthermore, as shown in Figure 3, the PLS-R correlation map indicated strong associations between specific volatile compounds, such as hexanal, nonanal, butane-2,3-diol, and octanal, and sensory attributes found in long-term-aged ganjang samples, such as savory, jochung, sour, spicy, burnt, and sweet. In contrast, ketone compounds, such as butane-2,3-dione, 2,3,5,6-tetramethylpyrazine, butanoic acid, 2-methylbutan-1-ol, and 3-methylbutanoic acid, showed high correlations with negative odor and flavor attributes in samples A3 and B3, which exhibited lower consumer acceptability. These findings highlight the complex relationship between specific volatile compounds and sensory characteristics that influence consumer preferences for aged ganjang, emphasizing the critical role of changes in the volatile profile during aging.

## 4. Conclusions

This study comprehensively investigated the impact of the aging period and manufacturer on the physicochemical properties, free amino acid profiles, volatile compounds, and sensory characteristics of ganjang. Although there are concerns that protein-rich foods may accumulate harmful substances, such as biogenic amines, during long-term fermentation, the ganjang samples used in this study were commercially available products that had undergone quality control certification. These findings demonstrate that aging plays a crucial role in influencing the quality and consumer acceptability of ganjang by affecting key attributes such as moisture content, protein content, acidity, and salinity. Prolonged aging results in decreased moisture and pH levels and increased total solids, proteins, crude fat, and acidity. These changes contributed to the overall taste and mouthfeel, making the aged samples exhibit more desirable flavor profiles.

Notably, the content of free amino acids, including glutamic and aspartic acids, increases with age, enhancing umami and sweetness, which positively impacts consumer preferences. Additionally, aldehydes such as 2-methylbutanal and 3-methylbutanal were more prevalent in the long-term aged samples, contributing to the complex aroma profile. Sensory evaluations revealed that aged ganjang samples were more acceptable to consumers and were characterized by attributes such as viscosity, color intensity, savory taste, and burnt flavor. In contrast, samples with shorter aging periods showed negative sensory traits such as bitterness and moldy odors, leading to lower acceptability.

In conclusion, this study highlights the importance of aging and manufacturing processes in optimizing the flavor and quality of ganjang, with long-term aging playing a key role in developing a rich umami taste and complex aroma profile favored by consumers. However, the relationship between aging time, flavor enhancement, and the potential accumulation of harmful substances is a complex and significant area that requires further investigation.

## Figures and Tables

**Table 1:** Sample code and information of ganjang samples examined in this study.

Sample Code	Location of Producers (Geographical Coordinates)	Producer	Aging Period (Year)	Production Year
A03	Chungcheong-do (36°53′ N, 127°15′ E)	Isaacfarm (A)	3	2020
A10			10	2013
B03	Jeolla-do (36°01′ N, 126°80′ E)	Onggojib (B)	3	2020
B10			10	2013
B15			15	2008
C03	Jeju-do (33°51′ N, 126°70′ E)	Doguri (C)	3	2020
C10			10	2013
C15			15	2008

**Table 2:** Proximate composition, physicochemical properties, and color values of ganjang with different aging periods.

Properties ^1^		A03	A10	B03	B10	B15	C03	C10	C15
Proximate Composition	Moisture (%) ^***^	65.0 ± 0.00 ^c^	56.7 ± 0.00 ^e^	72.1 ± 0.00 ^a^	57.9 ± 0.00 ^d^	52.0 ± 0.00 ^f^	65.2 ± 0.00 ^b^	43.3 ± 0.00 ^h^	44.9 ± 0.00 ^g^
	Total solids (%) ^***^	35.0 ± 0.00 ^f^	43.3 ± 0.00 ^d^	27.9 ± 0.00 ^h^	42.1 ± 0.00 ^e^	48.0 ± 0.00 ^c^	34.8 ± 0.00 ^g^	56.7 ± 0.00 ^a^	55.1 ± 0.00 ^b^
	Ash (%) ^***^	24.1 ± 0.00 ^b^	22.8 ± 0.00 ^e^	18.4 ± 0.00 ^g^	23.5 ± 0.00 ^d^	22.1 ± 0.00 ^f^	25.0 ± 0.00 ^a^	23.7 ± 0.00 ^c^	22.8 ± 0.00 ^e^
	Protein (%) ^***^	5.7 ± 0.00 ^f^	11.5 ± 0.00 ^c^	5.8 ± 0.00 ^f^	10.0 ± 0.00 ^e^	11.9 ± 0.00 ^b^	4.9 ± 0.00 ^g^	11.1 ± 0.00 ^d^	13.0 ± 0.00 ^a^
	Crude fat (%) ^***^	0.53 ± 0.00 ^c^	0.88 ± 0.00 ^a^	0.49 ± 0.00 ^cd^	0.89 ± 0.00 ^a^	0.64 ± 0.00 ^bc^	0.38 ± 0.00 ^d^	0.75 ± 0.00 ^ab^	0.83 ± 0.00 ^a^
Color Properties	L* ^***^	68.0 ± 0.32 ^abcd^	68.2 ± 0.46 ^abc^	68.5 ± 1.27 ^ab^	67.6 ± 0.01 ^bcd^	69.2 ± 0.03 ^a^	66.9 ± 0.01 ^cd^	67.6 ± 0.01 ^bcd^	66.7 ± 0.01 ^d^
	a* ^***^	0.04 ± 0.02 ^de^	0.04 ± 0.01 ^de^	0.52 ± 0.05 ^a^	0.08 ± 0.02 ^d^	−0.06 ± 0.02 ^f^	0.13 ± 0.01 ^c^	0.01 ± 0.00 ^e^	0.18 ± 0.01 ^b^
	B* ^***^	−0.20 ± 0.12 ^c^	−0.22 ± 0.13 ^c^	0.24 ± 0.14 ^a^	0.20 ± 0.03 ^a^	−0.36 ± 0.02 ^c^	0.04 ± 0.01 ^b^	−0.38 ± 0.01 ^c^	0.33 ± 0.02 ^a^
	C* ^***^	0.06 ± 0.03 ^d^	1.14 ± 0.00 ^a^	0.64 ± 0.06 ^b^	0.34 ± 0.10 ^c^	0.98 ± 0.06 ^a^	0.14 ± 0.02 ^d^	0.54 ± 0.16 ^b^	0.59 ± 0.11 ^b^
	h° ^***^	30.3 ± 22.49 ^a^	79.9 ± 0.86 ^a^	26.0 ± 6.15 ^b^	62.7 ± 2.21 ^a^	89.4 ± 0.53 ^a^	36.5 ± 6.88 ^b^	68.2 ± 2.37 ^a^	69.0 ± 16.69 ^a^
Salinity (%) ^***^		21.3 ± 0.11 ^b^	19.0 ± 0.07 ^e^	17.5 ± 0.08 ^f^	20.1 ± 0.17 ^c^	19.6 ± 0.11 ^d^	23.0 ± 0.04 ^a^	18.8 ± 0.07 ^e^	17.1 ± 0.04 ^g^
pH ^***^		4.75 ± 0.09 ^d^	4.85 ± 0.09 ^d^	6.37 ± 0.10 ^a^	5.69 ± 0.01 ^c^	5.92 ± 0.00 ^b^	5.92 ± 0.09 ^b^	4.55 ± 0.02 ^e^	4.52 ± 0.18 ^e^
Acidity ^2^ (%) ^***^		1.79 ± 0.04 ^f^	6.77 ± 0.29 ^b^	1.35 ± 0.04 ^f^	5.21 ± 0.25 ^d^	5.69 ± 0.26 ^c^	3.26 ± 0.15 ^e^	6.75 ± 0.30 ^b^	8.19 ± 0.37 ^b^

^1^ All values are presented as mean ± standard deviation of three replications; ^2^ Acidity (g lactic acid/100 mL). ^***^ represents a significant difference among the samples at *p* < 0.001. ^abcdefgh^ Different letters within the same row represent significant differences at *p* < 0.05 by SNK multiple comparison test.

**Table 3:** Amino acids contents in ganjang with different aging periods.

Amino Acids (mg/100 g) ^1^	A03	A10	B03	B10	B15	C03	C10	C15
Alanine ***	239.0 ± 12.09 ^g^	467.6 ± 0.63 ^d^	603.3 ± 16.96 ^c^	382.0 ± 2.07 ^e^	754.7 ± 4.12 ^b^	326.9 ± 2.82 ^f^	774.1 ± 7.10 ^b^	880.8 ± 0.92 ^a^
Anserine ***	213.4 ± 0.22 ^d^	338.8 ± 6.51 ^c^	99.6 ± 6.17 ^f^	231.5 ± 15.12 ^d^	232.9 ± 4.18 ^d^	150.7 ± 4.93 ^e^	430.4 ± 5.35 ^a^	389.0 ± 0.94 ^b^
Arginine ***	11.8 ± 0.43 ^e^	65.6 ± 0.24 ^a^	0.9 ± 0.00 ^f^	19.3 ± 0.12 ^d^	65.6 ± 0.33 ^a^	12.1 ± 0.26 ^e^	23.9 ± 0.16 ^c^	31.7 ± 0.01 ^b^
Aspartic acid ***	318.2 ± 16.22 ^b^	649.8 ± 2.00 ^a^	30.4 ± 0.50 ^f^	283.1 ± 0.86 ^c^	642.5 ± 3.29 ^a^	76.2 ± 0.66 ^e^	227.8 ± 1.65 ^d^	325.5 ± 0.07 ^b^
a-Amino adipic acid ***	29.0 ± 1.35 ^g^	65.9 ± 0.35 ^c^	39.8 ± 0.56 ^g^	53.2 ± 0.20 ^e^	59.3 ± 0.38 ^d^	67.5 ± 0.81 ^c^	86.2 ± 0.74 ^a^	72.8 ± 0.11 ^b^
a-Aminobutyric acid ***	9.3 ± 0.73 ^e^	21.6 ± 0.15 ^d^	151.9 ± 4.14 ^a^	24.2 ± 0.20 ^d^	14.5 ± 0.16 ^e^	54.6 ± 0.50 ^c^	62.2 ± 0.36 ^b^	49.9 ± 0.13 ^c^
β-Alanine ***	33.9 ± 2.25 ^e^	72.5 ± 0.32 ^b^	8.0 ± 0.06 ^g^	102.3 ± 0.40 ^a^	67.8 ± 0.11 ^c,d^	11.7 ± 0.12 ^f^	69.0 ± 0.58 ^c^	65.6 ± 0.20 ^d^
Citrulline ***	102.8 ± 5.17 ^c^	227.3 ± 0.51 ^a^	13.4 ± 0.28 ^f^	95.0 ± 0.71 ^d^	180.1 ± 0.84 ^b^	14.5 ± 0.16 ^f^	45.1 ± 0.51 ^e^	49.9 ± 0.11 ^e^
Cysteine ***	n.d.	n.d.	n.d.	n.d.	n.d.	3.6 ± 0.01 ^a^	n.d.	n.d.
Glutamic acid ***	600.9 ± 30.78 ^f^	1275.9 ± 3.55 ^b^	727.1 ± 19.50 ^e^	1075.4 ± 4.66 ^c^	1339.4 ± 8.00 ^a^	226.5 ± 2.23 ^g^	841.1 ± 6.42 ^d^	1070.3 ± 1.33 ^c^
Glycine ***	115.9 ± 8.63 ^e^	211.6 ± 0.52 ^c^	149.5 ± 4.20 ^d^	144.2 ± 0.66 ^d^	337.3 ± 4.37 ^a^	100.5 ± 0.93 ^f^	216.5 ± 1.53 ^c^	239.3 ± 0.18 ^b^
GABA ***	8.7 ± 0.46 ^g^	29.3 ± 0.04 ^e^	90.1 ± 2.44 ^d^	19.5 ± 0.01 ^f^	15.9 ± 0.01 ^f,g^	323.3 ± 3.29 ^b^	444.6 ± 4.34 ^a^	280.7 ± 0.06 ^c^
Histidine ***	79.1 ± 4.13 ^d^	151.1 ± 0.41 ^b^	26.6 ± 0.69 ^f^	83.8 ± 0.26 ^c,d^	180.1 ± 1.22 ^a^	13.7 ± 0.13 ^g^	34.3 ± 0.38 ^e^	87.7 ± 0.12 ^c^
Isoleucine ***	216.0 ± 11.73 ^f^	344.4 ± 1.71 ^d^	209.1 ± 5.34 ^f^	246.0 ± 1.10 ^e^	454.8 ± 3.41 ^a^	165.0 ± 2.30 ^g^	367.1 ± 1.76 ^c^	422.4 ± 1.05 ^b^
Leucine ***	327.4 ± 17.31 ^e^	516.4 ± 1.22 ^c^	311.7 ± 8.46 ^f^	387.4 ± 1.71 ^d^	616.7 ± 3.45 ^d^	279.4 ± 2.71 ^g^	608.9 ± 4.93 ^b^	677.6 ± 0.24 ^a^
Lysine ***	319.3 ± 16.14 ^g^	655.5 ± 1.69 ^d^	380.0 ± 10.64 ^f^	471.3 ± 1.13 ^e^	700.3 ± 4.61 ^c^	389.9 ± 3.85 ^f^	834.8 ± 6.54 ^b^	879.8 ± 1.14 ^a^
Methionine ***	24.1 ± 1.29 ^h^	38.7 ± 0.16 ^f^	46.8 ± 1.26 ^d^	27.3 ± 0.16 ^g^	94.2 ± 0.67 ^a^	43.0 ± 0.67 ^e^	78.2 ± 0.49 ^b^	75.0 ± 0.10 ^c^
Ornithine ***	61.9 ± 3.10 ^e^	147.3 ± 0.33 ^c^	14.7 ± 0.29 ^f^	122.2 ± 0.46 ^d^	373.4 ± 2.15 ^a^	323.4 ± 2.95 ^b^	373.8 ± 2.62 ^a^	318.3 ± 0.37 ^b^
ο-Phosphoethanolamine ***	17.5 ± 0.47 ^f^	35.2 ± 0.53 ^b^	n.d.	42.9 ± 0.47 ^a^	21.7 ± 0.16 ^e^	7.8 ± 0.13 ^h^	26.8 ± 0.40 ^d^	33.3 ± 0.26 ^c^
Phenylalanine ***	169.4 ± 8.99 ^g^	320.3 ± 1.41 ^d^	184.3 ± 5.10 ^f^	223.1 ± 0.97 ^e^	414.0 ± 1.99 ^a^	169.3 ± 1.55 ^g^	340.0 ± 1.86 ^c^	375.6 ± 0.34 ^b^
Proline ***	256.7 ± 3.25 ^e^	466.7 ± 0.71 ^c^	192.1 ± 4.51 ^f^	352.0 ± 3.91 ^d^	501.0 ± 3.73 ^b^	135.0 ± 0.58 ^g^	371.1 ± 14.19 ^d^	522.5 ± 1.37 ^a^
ο-Phosphoserine ***	28.3 ± 1.13 ^f^	67.6 ± 0.65 ^b^	18.6 ± 0.15 ^g^	58.5 ± 0.20 ^c^	53.4 ± 0.30 ^d^	14.0 ± 0.10 ^h^	50.7 ± 0.62 ^e^	69.9 ± 0.28 ^a^
Serine ***	234.9 ± 11.76 ^f^	428.3 ± 1.29 ^b^	5.4 ± 0.08 ^f^	245.9 ± 0.93 ^d^	605.7 ± 3.74 ^a^	74.2 ± 0.63 ^e^	302.0 ± 2.35 ^c^	440.1 ± 0.39 ^b^
Taurine ***	16.5 ± 0.22 ^d^	22.9 ± 0.05 ^b^	15.1 ± 0.11 ^f^	24.2 ± 0.28 ^a^	24.6 ± 0.06 ^a^	9.2 ± 0.05 ^e^	19.3 ± 0.21 ^c^	22.7 ± 0.83 ^b^
Threonine ***	193.4 ± 9.83 ^f^	345.5 ± 0.75 ^c^	10.5 ± 0.41 ^h^	212.6 ± 0.97 ^e^	438.7 ± 2.95 ^a^	96.3 ± 0.85 ^g^	290.6 ± 1.98 ^d^	370.9 ± 0.35 ^b^
Tyrosine ***	72.7 ± 4.06 ^f^	163.9 ± 0.54 ^a^	34.6 ± 0.97 ^g^	149.8 ± 0.54 ^b^	76.1 ± 0.46 ^f^	107.9 ± 1.22 ^d^	88.3 ± 0.55 ^e^	142.6 ± 0.32 ^c^
Valine ***	245.3 ± 12.55 ^e^	434.8 ± 1.02 ^c^	249.5 ± 6.57 ^e^	309.0 ± 1.35 ^d^	558.2 ± 6.38 ^a^	195.4 ± 2.65 ^f^	447.3 ± 3.64 ^c^	529.8 ± 1.28 ^b^
Total	3945.7	7564.4	3613.0	5385.8	8822.9	3391.7	7454.0	8423.6

^1^ All values are presented as mean ± standard deviation of two replications. GABA, 4-aminobutyric acid. *** represents a significant difference among the samples at *p* < 0.001. Different superscript letters within the same row represent significant differences at *p* < 0.05 in the SNK multiple comparison test. n.d.: Not detected.

**Table 4:** Volatile compounds in ganjang with different aging periods.

Volatile Compounds (mg/mL) ^1^	A03	A10	B03	B10	B15	C03	C10	C15	RT	VIP
**alcohols**										
ethanol	170.6 ± 4.70 ^b^	94.4 ± 3.43 ^d^	227.4 ± 13.12 ^a^	n.d.	110.5 ± 9.67 ^c^	n.d.	n.d.	n.d.	3.24	1.18
2-methyl-3-sulfanylpropan-1-ol	101.0 ± 0.21 ^a^	n.d.	98.9 ± 1.41 ^a^	n.d.	75.3 ± 3.28 ^b^	n.d.	n.d.	n.d.	6.22	1.19
2-methylbutan-1-ol	401.9 ± 251.74 ^a^	78.0 ± 1.97 ^b^	329.2 ± 14.41 ^a^	79.9 ± 1.30 ^b^	136.3 ± 9.99 ^b^	n.d.	n.d.	n.d.	9.15	0.99
3-methylbutan-1-ol	332.4 ± 18.05 ^b^	74.8 ± 3.74 ^d^	517.2 ± 34.55 ^a^	83.2 ± 1.15 ^d^	148.2 ± 14.34 ^c^	n.d.	n.d.	n.d.	9.17	1.26
octan-3-ol	n.d.	n.d.	67.8 ± 0.99 ^b^	n.d.	74.0 ± 2.69 ^a^	n.d.	n.d.	n.d.	14.28	1.25
oct-1-en-3-ol	69.3 ± 1.05 ^b^	n.d.	81.4 ± 2.95 ^b^	n.d.	258.9 ± 31.25 ^a^	n.d.	n.d.	n.d.	15.37	1.18
nonan-2-ol	247.2 ± 1.84 ^a^	90.9 ± 5.78 ^b^	n.d.	n.d.	n.d.	n.d.	n.d.	n.d.	16.40	0.27
butane-2,3-diol	161.371 ± 6.46 ^d^	209.29 ± 38.92 ^c,d^	141.53 ± 2.68 ^d^	162.39 ± 5.24 ^d^	143.83 ± 3.90 ^d^	256.79 ± 75.52 ^b,c^	389.12 ± 48.83 ^a^	290.60 ± 22.89 ^b^	16.70	0.80
furan-2-ylmethanol	n.d.	96.8 ± 9.89 ^d^	74.9 ± 2.89 ^e^	102.5 ± 3.50 ^d^	237.3 ± 14.70 ^a^	n.d.	120.6 ± 2.33 ^c^	136.4 ± 1.11 ^b^	17.86	1.19
2-phenylethanol	89.9 ± 3.14 ^c^	n.d.	506.0 ± 27.82 ^a^	n.d.	135.1 ± 7.49 ^b^	n.d.	n.d.	n.d.	19.82	1.24
**aldehydes**										
2-Methylpropanal	48.4 ± 21.84 ^h^	600.4 ± 63.87 ^d^	344.9 ± 31.36 ^f^	525.4 ± 40.21 ^e^	1493.9 ± 55.13 ^a^	233.0 ± 21.07 ^g^	793.6 ± 14.08 ^c^	899.4 ± 10.99 ^b^	2.09	1.19
2-methylbutanal	201.2 ± 16.87 ^c^	959.5 ± 109.05 ^b^	1279.3 ± 1445.21 ^a^	873.0 ± 131.85 ^b^	1602.9 ± 41.85 ^a^	226.3 ± 31.45 ^c^	1253.6 ± 78.16 ^a^	1452.0 ± 72.47 ^a^	2.91	0.66
3-methylbutanal	1196.4 ± 68.18 ^c^	3553.5 ± 452.01 ^b^	3127.5 ± 765.02 ^b^	3740.2 ± 181.03 ^b^	4644.7 ± 939.04 ^a^	528.5 ± 161.00 ^c^	5417.6 ± 254.35 ^a^	5402.0 ± 205.95 ^a^	2.95	0.78
hexanal	72.2 ± 5.04	84.2 ± 18.70	71.3 ± 4.14	76.6 ± 8.28	70.6 ± 3.24	79.0 ± 12.58	72.3 ± 5.38	71.9 ± 6.25	5.77	0.46
octanal	65.8 ± 1.59	66.7 ± 3.31	66.6 ± 2.52	66.9 ± 4.12	71.4 ± 6.28	69.7 ± 4.44	68.8 ± 5.41	67.6 ± 0.84	11.23	0.51
nonanal	n.d.	80.2 ± 11.14 ^a^	75.5 ± 3.58 ^a^	82.7 ± 11.11 ^a^	n.d.	86.9 ± 11.62 ^a^	83.1 ± 13.56 ^a^	81.4 ± 4.97 ^a^	14.11	0.62
benzaldehyde	93.9 ± 2.06 ^d^	158.8 ± 24.20 ^b^	130.2 ± 6.64 ^c^	189.5 ± 7.89 ^a^	220.1 ± 26.84 ^a^	81.1 ± 5.67 ^d^	213.2 ± 5.72 ^a^	204.0 ± 5.09 ^a^	16.33	1.00
**ketones**										
butan-2-one	90.3 ± 3.63 ^b^	200.0 ± 19.44 ^b^	304.8 ± 237.40 ^b^	170.3 ± 9.83 ^b^	595.0 ± 11.55 ^a^	151.7 ± 4.87 ^b^	238.6 ± 5.00 ^b^	245.5 ± 7.24 ^b^	2.77	1.06
3-methylbutan-2-one	n.d.	n.d.	n.d.	n.d.	82.4 ± 5.47 ^a^	76.3 ± 7.23 ^b^	n.d.	n.d.	3.10	0.91
butane-2,3-dione	74.2 ± 6.75 ^d^	83.0 ± 1.00 ^c,d^	180.6 ± 5.29 ^a^	77.8 ± 0.49 ^c,d^	82.1 ± 1.39 ^c,d^	195.4 ± 9.89 ^a^	87.0 ± 1.42 ^c^	79.0 ± 2.63 ^c,d^	3.78	1.07
4-methylhexan-2-one	n.d.	70.3 ± 0.86 ^b^	n.d.	n.d.	83.1 ± 1.47 ^a^	n.d.	n.d.	n.d.	6.67	1.15
5-methylhexan-2-one	n.d.	73.8 ± 1.97 ^b^	n.d.	n.d.	98.7 ± 0.72 ^a^	n.d.	n.d.	n.d.	7.26	1.17
heptane-2,3-dione	n.d.	n.d.	n.d.	n.d.	n.d.	n.d.	n.d.	n.d.	7.52	0.43
3-hydroxybutan-2-one	71.4 ± 1.92 ^c^	74.6 ± 4.15 ^c^	67.2 ± 1.39 ^c^	67.4 ± 0.52 ^c^	n.d.	174.1 ± 12.06 ^a^	90.4 ± 1.57 ^b^	75.1 ± 0.09 ^c^	11.08	0.69
1-hydroxypropan-2-one	n.d.	70.3 ± 4.10 ^b^	n.d.	74.2 ± 0.90 ^b^	84.9 ± 3.02 ^a^	65.5 ± 0.54 ^c^	71.3 ± 0.88 ^b^	72.6 ± 1.15 ^b^	11.45	1.25
nonan-2-one	1063.2 ± 25.86 ^a^	77.5 ± 2.16 ^b^	n.d.	n.d.	n.d.	n.d.	n.d.	n.d.	14.01	0.34
undecan-2-one	79.4 ± 0.99 ^b^	n.d.	n.d.	n.d.	n.d.	94.2 ± 5.37 ^a^	67.6 ± 0.41 ^c^	n.d.	17.29	0.36
**pyrazines**										
2-methylpyrazine	n.d.	n.d.	70.1 ± 1.89 ^b^	65.8 ± 0.56 ^c^	78.7 ± 2.86 ^a^	n.d.	n.d.	n.d.	10.58	1.24
2,5-dimethylpyrazine	n.d.	n.d.	81.0 ± 2.29 ^a^	n.d.	70.4 ± 1.09 ^b^	n.d.	n.d.	n.d.	12.17	1.37
2,6-dimethylpyrazine	66.6 ± 0.40 ^c^	74.7 ± 3.24 ^b^	78.8 ± 0.34 ^b^	76.9 ± 2.92 ^b^	125.1 ± 6.54 ^a^	n.d.	68.0 ± 1.67 ^c^	74.7 ± 1.69 ^b^	12.35	1.21
2-ethyl-6-methylpyrazine	n.d.	69.7 ± 2.54 ^b^	72.1 ± 1.71 ^b^	66.0 ± 0.79 ^b^	124.2 ± 6.42 ^a^	n.d.	67.6 ± 1.22 ^b^	70.3 ± 0.53 ^b^	13.93	1.21
2,3,5-trimethylpyrazine	n.d.	n.d.	112.7 ± 2.48 ^a^	n.d.	68.5 ± 0.16 ^c^	76.9 ± 2.38 ^b^	67.5 ± 0.33 ^c^	69.5 ± 0.89 ^c^	14.42	1.30
2-ethyl-3,5-dimethylpyrazine	n.d.	n.d.	71.1 ± 1.29 ^a^	n.d.	n.d.	n.d.	n.d.	n.d.	15.54	1.26
2,3,5,6-tetramethylpyrazine	67.1 ± 0.28 ^c^	n.d.	130.6 ± 5.21 ^a^	n.d.	n.d.	75.9 ± 1.81 ^b^	n.d.	n.d.	15.76	1.10
**hydrocarbons**										
1,4-xylene	70.7 ± 0.93 ^a^	71.9 ± 1.13 ^a^	67.0 ± 0.41 ^b^	67.2 ± 1.51 ^b^	n.d.	n.d.	n.d.	n.d.	7.10	0.14
2,4-dimethylpentane	n.d.	n.d.	73.6 ± 1.34 ^a^	n.d.	n.d.	n.d.	n.d.	n.d.	7.50	1.18
2-heptyl-1,3-dioxepane	n.d.	126.8 ± 10.86 ^a^	n.d.	n.d.	n.d.	n.d.	75.5 ± 1.40 ^b^	74.5 ± 0.70 ^b^	7.54	0.99
2-pentadecyl-1,3-dioxepane	n.d.	77.1 ± 5.34 ^a^	n.d.	n.d.	n.d.	n.d.	72.8 ± 1.05 ^b^	67.8 ± 0.44 ^c^	7.72	1.12
1-methyl-4-prop-1-en-2 -ylcyclohexene	65.8 ± 0.51	78.3 ± 20.73	108.8 ± 66.44	78.1 ± 13.58	89.1 ± 35.52	82.0 ± 26.72	80.2 ± 22.60	66.3 ± 0.98	8.67	0.53
2-bromo-2-methylpropane	n.d.	n.d.	n.d.	n.d.	156.5 ± 2.97 ^a^	n.d.	n.d.	n.d.	9.62	1.17
**FAs**										
4-oxoheptanedioic acid	n.d.	119.7 ± 3.71 ^a^	n.d.	n.d.	n.d.	n.d.	n.d.	n.d.	5.10	0.78
4-oxoheptanedioic acid	n.d.	94.6 ± 1.65 ^a^	n.d.	n.d.	n.d.	n.d.	n.d.	n.d.	9.01	0.78
acetic acid	119.3 ± 7.49 ^d^	261.3 ± 72.54 ^d^	97.6 ± 10.83 ^d^	1018.4 ± 11.42 ^b^	590.1 ± 121.29 ^c^	1622.8 ± 312.25 ^a^	1487.7 ± 65.33 ^a^	1431.2 ± 115.06 ^a^	15.28	0.95
2-methylpropanoic acid	84.7 ± 1.17 ^c,d^	72.8 ± 4.11 ^e^	98.6 ± 7.36 ^b^	92.3 ± 2.47 ^b,c^	218.1 ± 11.74 ^a^	79.1 ± 2.40 ^d,e^	n.d.	n.d.	16.91	0.82
butanoic acid	n.d.	n.d.	77.6 ± 4.20 ^b^	202.8 ± 8.97 ^a^	n.d.	n.d.	n.d.	n.d.	17.54	0.50
3-methylbutanoic acid	153.6 ± 11.52 ^a,b^	89.4 ± 12.39 ^b^	97.8 ± 80.65 ^b^	121.4 ± 4.75 ^a,b^	188.8 ± 39.77 ^a^	109.9 ± 4.87 ^b^	99.5 ± 3.71 ^b^	97.6 ± 2.67 ^b^	17.93	0.86
**Esters**										
ethyl acetate	76.9 ± 6.73 ^a,b^	n.d.	80.1 ± 2.95 ^a,b^	74.8 ± 7.20 ^b^	84.5 ± 2.53 ^a^	75.4 ± 1.51 ^b^	n.d.	n.d.	2.64	1.10
methyl 2-hydroxy-4-methylpentanoate	n.d.	n.d.	73.0 ± 3.13 ^a^	n.d.	n.d.	n.d.	n.d.	n.d.	15.66	1.15
S-ethyl hexanethioate	n.d.	n.d.	n.d.	n.d.	73.4 ± 1.31 ^a^	n.d.	n.d.	n.d.	17.12	1.17
Isobornyl acetate	n.d.	73.7 ± 8.91 ^a^	n.d.	n.d.	75.8 ± 10.36 ^a^	n.d.	n.d.	n.d.	17.17	0.43
methyl 2-hydroxybenzoate	129.4 ± 47.76	184.1 ± 52.71	121.1 ± 17.09	167.1 ± 30.87	160.0 ± 50.68	124.1 ± 30.56	122.3 ± 12.46	136.8 ± 42.50	18.90	0.61
**Nitriles/Sulfurs/Phenol**										
(methyldisulfanyl)methane	97.3 ± 4.63 ^c^	310.4 ± 22.00 ^a^	84.6 ± 8.13 ^c^	164.5 ± 10.82 ^b^	188.3 ± 32.92 ^b^	80.1 ± 7.86 ^c^	168.3 ± 9.67 ^b^	177.7 ± 26.82 ^b^	5.51	1.24
3-methylbutanenitrile	71.8 ± 0.38 ^c^	87.2 ± 0.42 ^b^	71.2 ± 2.45 ^c^	69.4 ± 1.29 ^c^	725.9 ± 16.60 ^a^	n.d.	n.d.	n.d.	6.75	1.17
(methyltrisulfanyl)methane	74.4 ± 2.3 ^d^	409.4 ± 30.70 ^a^	78.3 ± 8.57 ^d^	136.1 ± 9.02 ^b^	104.3 ± 14.23 ^c^	n.d.	109.8 ± 8.04 ^b,c^	134.6 ± 8.52 ^b^	13.54	0.98
2-methoxyphenol	n.d.	n.d.	88.2 ± 7.63 ^b^	n.d.	115.3 ± 6.32 ^a^	n.d.	n.d.	n.d.	19.46	1.26

^1^ All values are presented as mean ± standard deviation of three replications. ^abcdefgh^ Different letters within the same row represent significant differences at *p* < 0.001 by SNK multiple comparison test. n.d.: Not detected; RT: Retention Time; VIP: Variable Importance in ProjectionIn contrast, doenjang and moldy odors tended to decrease, indicating a reduction in specific odors associated with meju as the ganjang aged. Among the attributes associated with taste/flavor and mouthfeel, only sweetness, doenjang, and thickness showed significant differences in ganjang samples with different aging periods.

**Table 5:** Descriptive characteristics of ganjang samples.

Descriptive Attributes ^1^	A03	A10	B03	B10	B15	C03	C10	C15
**Appearance**								
Viscosity ***	7.07 ± 0.87 ^d^	9.84 ± 1.31 ^a,b^	5.65 ± 1.47 ^e^	9.26 ± 1.03 ^b,c^	8.78 ± 1.01 ^c^	6.10 ± 1.36 ^e^	9.31 ± 1.08 ^b,c^	10.12 ± 1.58 ^a^
Precipitates ***	2.02 ± 1.53 ^a^	1.86 ± 1.30 ^a,b^	0.81 ± 0.50 ^c^	1.61 ± 1.24 ^a,b^	1.19 ± 0.78 ^b,c^	1.09 ± 0.98 ^b,c^	1.42 ± 0.92 ^a,b,c^	1.87 ± 1.65 ^a,b^
Degree of color ***	8.49 ± 1.02 ^b^	11.49 ± 1.20 ^a^	5.60 ± 1.72 ^c^	11.22 ± 1.17 ^a^	10.67 ± 1.03 ^a^	6.13 ± 1.93 ^c^	11.18 ± 1.10 ^a^	11.54 ± 1.21 ^a^
Greenish brown color ***	2.57 ± 1.69 ^c^	1.14 ± 0.83 ^d^	3.94 ± 2.27 ^b^	1.39 ± 1.16 ^d^	1.37 ± 1.04 ^d^	5.23 ± 2.27 ^a^	1.26 ± 0.84 ^d^	1.14 ± 0.85 ^d^
**Odor**								
Jocheong ***	3.83 ± 1.36 ^c,d^	5.04 ± 1.58 ^a,b^	2.49 ± 1.00 ^e^	4.62 ± 1.49 ^a,b,c^	3.47 ± 1.59 ^d^	4.37 ± 1.48 ^b,c^	5.02 ± 1.81 ^a,b^	5.36 ± 1.41 ^a^
Sour	3.42 ± 1.34 ^a^	4.03 ± 1.37 ^a^	3.70 ± 1.53 ^a^	4.04 ± 1.58 ^a^	3.66 ± 1.51 ^a^	3.88 ± 1.66 ^a^	3.80 ± 1.66 ^a^	4.16 ± 1.93 ^a^
Savory **	3.66 ± 1.06 ^b^	4.36 ± 1.20 ^a^	4.10 ± 1.06 ^a,b^	4.46 ± 1.33 ^a^	3.67 ± 1.30 ^b^	4.21 ± 1.16 ^a,b^	4.56 ± 1.45 ^a^	4.53 ± 1.61 ^a^
Doenjang ***	4.31 ± 1.77 ^b^	4.10 ± 1.12 ^b^	6.16 ± 1.78 ^a^	4.13 ± 1.16 ^b^	3.99 ± 1.37 ^b^	4.62 ± 1.49 ^b^	4.11 ± 1.31 ^b^	4.10 ± 1.30 ^b^
Burnt ***	2.21 ± 1.29 ^b^	3.56 ± 1.80 ^a^	2.23 ± 1.28 ^b^	3.76 ± 2.03 ^a^	3.63 ± 2.32 ^a^	2.26 ± 1.21 ^b^	3.37 ± 1.87 ^a^	3.59 ± 1.93 ^a^
Spicy *	2.48 ± 0.95 ^b,c,d^	3.02 ± 1.11 ^a,b^	2.40 ± 0.86 ^c,d^	2.96 ± 1.42 ^a,b,c^	2.74 ± 0.98 ^a,b,c,d^	2.32 ± 0.80 ^d^	2.84 ± 1.35 ^a,b,c,d^	3.09 ± 1.37 ^a^
Moldy ***	3.78 ± 1.06 ^c^	3.80 ± 0.90 ^c^	4.77 ± 1.23 ^a^	3.89 ± 1.13 ^c^	4.67 ± 1.64 ^a,b^	4.15 ± 1.13 ^a,b,c^	3.80 ± 1.03 ^c^	4.03 ± 0.94 ^b,c^
Fish sauce	3.11 ± 1.53 ^a^	3.35 ± 1.34 ^a^	3.85 ± 1.77 ^a^	3.71 ± 1.40 ^a^	3.38 ± 1.43 ^a^	3.39 ± 1.64 ^a^	3.23 ± 1.37 ^a^	3.42 ± 1.41 ^a^
**Taste/flavor**								
Sweetness **	3.16 ± 0.69 ^a,b^	3.38 ± 0.67 ^a,b^	2.93 ± 0.85 ^b^	3.39 ± 0.69 ^a,b^	3.34 ± 0.71 ^a,b^	2.84 ± 0.85 ^c^	3.13 ± 0.81 ^a,b^	3.46 ± 0.67 ^a^
Sourness	3.47 ± 0.80 ^a^	3.72 ± 0.84 ^a^	3.33 ± 0.70 ^a^	3.64 ± 0.84 ^a^	3.47 ± 0.82 ^a^	3.67 ± 1.00 ^a^	3.73 ± 0.86 ^a^	3.72 ± 0.89 ^a^
Bitterness	2.92 ± 0.86 ^a^	3.15 ± 0.83 ^a^	2.84 ± 0.80 ^a^	3.05 ± 0.88 ^a^	2.85 ± 0.80 ^a^	3.11 ± 0.82 ^a^	3.15 ± 0.73 ^a^	3.08 ± 0.89 ^a^
Saltiness	10.08 ± 1.36 ^a^	10.32 ± 1.39 ^a^	10.14 ± 1.33 ^a^	10.14 ± 1.31 ^a^	9.84 ± 1.61 ^a^	10.47 ± 1.25 ^a^	10.41 ± 1.25 ^a^	10.33 ± 1.42 ^a^
Umami	4.98 ± 1.43 ^a^	5.29 ± 1.50 ^a^	4.88 ± 1.37 ^a^	5.05 ± 1.38 ^a^	5.03 ± 1.25 ^a^	4.80 ± 1.32 ^a^	5.14 ± 1.55 ^a^	5.34 ± 1.42 ^a^
Doenjang ***	4.00 ± 1.08 ^b^	4.05 ± 0.99 ^b^	5.09 ± 1.26 ^a^	3.92 ± 1.03 ^b^	3.86 ± 1.13 ^b^	3.89 ± 1.13 ^b^	3.91 ± 1.04 ^b^	3.95 ± 1.10 ^b^
Savory	4.28 ± 1.33 ^a^	4.82 ± 1.26 ^a^	4.50 ± 0.93 ^a^	4.69 ± 1.24 ^a^	4.30 ± 1.22 ^a^	4.20 ± 1.43 ^a^	4.71 ± 1.29 ^a^	4.58 ± 1.29 ^a^
Fish sauce	3.67 ± 1.53 ^a^	3.51 ± 1.29 ^a^	4.14 ± 1.46 ^a^	3.81 ± 1.17 ^a^	3.64 ± 1.33 ^a^	3.71 ± 1.74 ^a^	3.56 ± 1.50 ^a^	3.50 ± 1.26 ^a^
**Mouthfeel**								
Thickness ***	4.26 ± 1.38 ^b^	5.82 ± 1.67 ^a^	3.88 ± 1.08 ^b,c^	5.74 ± 1.40 ^a^	5.35 ± 1.58 ^a^	3.41 ± 1.14 ^c^	5.66 ± 1.78 ^a^	6.06 ± 1.61 ^a^
Spiciness	3.08 ± 1.57 ^a^	3.27 ± 1.50 ^a^	3.21 ± 1.55 ^a^	3.18 ± 1.47 ^a^	3.06 ± 1.41 ^a^	3.07 ± 1.41 ^a^	3.40 ± 1.70 ^a^	3.46 ± 1.60 ^a^
Astringent	4.02 ± 1.51 ^a^	4.01 ± 1.43 ^a^	3.99 ± 1.48 ^a^	3.91 ± 1.40 ^a^	3.89 ± 1.61 ^a^	4.08 ± 1.44 ^a^	4.09 ± 1.38 ^a^	3.99 ± 1.53 ^a^
**Aftertaste**								
Sweet	2.08 ± 0.56 ^a^	2.13 ± 0.48 ^a^	1.94 ± 0.50 ^a^	2.05 ± 0.40 ^a^	2.07 ± 0.51 ^a^	1.84 ± 0.43 ^a^	2.00 ± 0.51 ^a^	2.12 ± 0.42 ^a^
Sour	2.26 ± 0.63 ^a^	2.36 ± 0.64 ^a^	2.06 ± 0.54 ^a^	2.27 ± 0.52 ^a^	2.19 ± 0.46 ^a^	2.30 ± 0.56 ^a^	2.26 ± 0.66 ^a^	2.32 ± 0.68 ^a^
Bitter	3.16 ± 1.16 ^a^	3.40 ± 1.08 ^a^	3.09 ± 1.16 ^a^	3.41 ± 1.17 ^a^	3.15 ± 1.14 ^a^	3.39 ± 1.19 ^a^	3.47 ± 1.03 ^a^	3.44 ± 1.19 ^a^
Salty	7.47 ± 1.26 ^a^	7.52 ± 1.28 ^a^	7.24 ± 1.38 ^a^	7.54 ± 1.21 ^a^	7.37 ± 1.50 ^a^	7.71 ± 1.22 ^a^	7.68 ± 1.28 ^a^	7.52 ± 1.58 ^a^
Umami	3.80 ± 0.91 ^a^	3.95 ± 1.02 ^a^	3.81 ± 1.01 ^a^	3.93 ± 0.88 ^a^	3.88 ± 0.97 ^a^	3.59 ± 0.90 ^a^	3.91 ± 0.98 ^a^	3.89 ± 0.97 ^a^
Savory	3.51 ± 0.95 ^a^	3.86 ± 0.81 ^a^	3.90 ± 0.70 ^a^	3.81 ± 0.91 ^a^	3.62 ± 0.88 ^a^	3.48 ± 0.95 ^a^	3.79 ± 1.03 ^a^	3.78 ± 0.94 ^a^
**Aftertaste Mouthfeel**								
Tingling	4.20 ± 1.26 ^a^	4.71 ± 1.38 ^a^	4.18 ± 1.52 ^a^	4.64 ± 1.21 ^a^	4.66 ± 1.31 ^a^	4.37 ± 1.53 ^a^	4.64 ± 1.25 ^a^	4.64 ± 1.47 ^a^
Stinging	2.58 ± 0.94 ^a^	2.81 ± 1.02 ^a^	2.79 ± 1.16 ^a^	2.63 ± 0.99 ^a^	2.71 ± 1.08 ^a^	2.99 ± 1.17 ^a^	2.88 ± 1.32 ^a^	2.71 ± 1.14 ^a^
Astringent	3.88 ± 1.53 ^a^	3.87 ± 1.43 ^a^	3.82 ± 1.54 ^a^	3.93 ± 1.31 ^a^	4.04 ± 1.42 ^a^	3.99 ± 1.52 ^a^	3.98 ± 1.49 ^a^	4.11 ± 1.41 ^a^

^1^ All values are presented as mean ± standard deviation of three replications with 11 trained panelists. *, **, *** represents a significant difference among the samples at *p* < 0.05, *p* < 0.01 and *p* < 0.001 respectively. ^abcde^ Different letters within the same row represent significant differences at *p* < 0.05 by SNK multiple comparison test.

**Table 6:** Consumer acceptability of ganjang samples.

Acceptability ^1^	A03	A10	B03	B10	B15	C03	C10	C15
Overall ***	6.10 ± 1.53 ^a,b^	6.39 ± 1.64 ^a^	4.61 ± 1.88 ^c^	5.76 ± 1.71 ^a,b^	5.66 ± 2.10 ^b^	4.66 ± 1.72 ^c^	6.10 ± 1.44 ^a,b^	6.13 ± 1.50 ^a,b^
Appearance ***	6.62 ± 1.35 ^a^	6.16 ± 1.74 ^a,b^	5.71 ± 1.63 ^b^	6.33 ± 1.61 ^a^	6.63 ± 1.47 ^a^	4.64 ± 1.74 ^c^	6.35 ± 1.43 ^a^	6.09 ± 1.71 ^a,b^
Odor/Aroma ***	6.02 ± 1.75 ^a^	6.25 ± 1.87 ^a^	3.53 ± 1.88 ^c^	4.88 ± 2.10 ^b^	5.02 ± 2.34 ^b^	4.53 ± 1.77 ^b^	6.03 ± 1.53 ^a^	6.35 ± 1.48 ^a^
Taste/Flavor ***	5.79 ± 1.94 ^a^	6.34 ± 1.81 ^a^	4.64 ± 1.90 ^b^	5.62 ± 1.91 ^a^	5.75 ± 2.14 ^a^	4.52 ± 2.02 ^b^	5.98 ± 1.68 ^a^	6.08 ± 1.81 ^a^
Mouthfeel ***	6.02 ± 1.54 ^a^	6.16 ± 1.66 ^a^	5.16 ± 1.72 ^b^	5.84 ± 1.73 ^a^	5.84 ± 1.81 ^a^	5.00 ± 1.76 ^b^	6.04 ± 1.49 ^a^	6.20 ± 1.44 ^a^

^1^ All values are presented as mean ± standard deviation of 102 consumers. *** represents a significant difference among the samples at *p* < 0.001. ^abc^ Different letters within the same row represent significant differences at *p* < 0.05 by SNK multiple comparison test.

## Data Availability

The original contributions presented in the study are included in the article/Supplementary Material, further inquiries can be directed to the corresponding authors.

## Supplementary Materials

The following supporting information can be downloaded from https://www.mdpi.com/article/10.3390/foods13203326/s1. Table S1: Descriptive attributes, definitions, and reference materials and reference intensity of nine soy sauce samples with different aging periods.
