# Supplementary material for: Physicochemical Attributes Related to Sensory Characteristics of Long-Term Aged Korean Traditional Soy Sauce (Ganjang)
Source: Foods. 2024 Oct 20;13(20):3326. doi: 10.3390/foods13203326 (PMC11507495; doi:10.3390/foods13203326)
Supplement: Supplementary file 1 [file foods-13-03326-s001.zip › foods-3224803-supplementary.pdf]

Supplementary Table S1. Descriptive attributes, definitions, and reference materials and reference intensity of nine soy sauce samples with different aging periods.

Attributes	Definition	Reference material	Reference intensity
Appearance			
Viscosity	Viscosity when gently agitated	40% Sucrose(10ml, CJ Cheiljedang Co., Seoul, Korea) solution containing 0.1% edible dyes(Eltin food coloring black(Lgreen Tech., Daejun, Korea)	7.3
Precipitates	Amount of visible sediment when gently shaken and tilted	10% (w/w)roasted soybean flour in water(10ml)	5.2
Color intensity	Degree of overall color intensity	40% Sucrose(10ml, CJ Cheiljedang Co., Seoul, Korea) solution containing 0.1% edible dyes (Eltin food coloring black(Lgreen Tech., Daejun, Korea)	9.5
Greenish brown	Degree of greenish-brown color intensity	Penton Color Book C#7769 (Pantone, NJ, USA)	9.9
Odor /Aroma			
Jochung	Sweet aroma associated with rice syrup (Jochung)	Rice syrup 10ml, (Pulmuone Co, Seoul, Korea.)	8.3
Sour	Sharp and pungent aroma associated with vinegar	50% brewing vinegar (10ml, Ottogi Co. Ltd., Seoul, Korea)	10.0
Savory	Nutty aroma from steamed beans	Cooked bean (5g, cooked at 121°C for 30 min).	8.8
Doenjang	Fermented aroma associated with fermented soybean paste (Doenjang)	10% Doenjang solution (10ml, Daesang Co., Seoul. Korea)	9.5
Burnt	Smoky odor associated with smoked tuna fish sauce	30% smoked tuna fish sauce solution (10ml, (Sajodaelim Co., Seoul, Korea)	10.0
Spicy	Spicy odor associated with hot pepper powder	5% red pepper powder solution(10ml)	8.1
Moldy	Moldy odor associated with mold or mushroom	root of brown enoki mushroom (2g)	8.3
Fish sauce	Odor associated with fermented anchovy fish sauce	50% anchovy fish sauce (10ml, CJ Cheiljedang Co., Seoul, Korea)	9.9
Taste/Flavor			
Sweet	Sucrose solution-related fundamental taste	1% Sucrose solution (10ml, CJ Cheiljedang Co., Seoul, Korea)	3.4
Sour	Citric acid solution-related fundamental taste	0.05% Citric acid solution(10ml, EdentownF&B, Incheon, Korea)	3.8
Bitter	Caffeine solution-related fundamental taste	2mM Caffeine Solution(10 ml, Guarana 10% extract powder, Cremar, Seoul, Korea)	3.4
Salty	Sodium chloride solution-related fundamental taste	10% NaCl solution(10ml, Morton iodized salt, Morton Salt, Inc., Chicago, IL, USA)	11.1

Umami	Mono sodium glutamate-related fundamental taste	0.2% MSG in 0.1% NaCl solution (10ml, Miwon, Daesang Co., Seoul, Korea)	7.1
Doenjang	Fermented food flavor related to fermented soybean paste (Doenjang)	10% Doenjang solution (10ml, Daesang Co., Seoul. Korea)	7.8
Savory	Savory flavor related to steamed beans	Cooked bean (5g, cooked at 121°C for 30 min).	9.5
Fish sauce	Salty and savory flavor associated with fermented anchovy sauce	50% anchovy fish sauce (10ml, CJ Cheiljedang Co., Seoul, Korea)	8.5
Mouthfeel			
Heavy mouthfeel	Degree of heavy and thick mouthfeel	Soy milk (10 ml, Maeil Dairies Co. Ltd. Seoul, Korea)	8.4
Spicy	Spicy and fiery mouthfeel associated with red pepper powder	5% red pepper powder solution(10ml)	9.2
Astringent	Astringent mouthfeel associated with alum solution	0.15% alum solution (10 ml, Alum, McCormick & Co., Inc., Baltimore, MD, USA)	6.3
After taste/flavor	A lingering aftertaste/flavor beyond 5 seconds after swallowing		
Sweet	sweetness related to sucrose after swallowing	1% Sucrose solution (10ml, CJ Cheiljedang Co., Seoul, Korea)	2.0
Sour	sourness related to citric acids after swallowing	0.05% Citric acid solution(10ml, EdentownF&B, Incheon, Korea)	2.5
Bitter	Caffeine solution-related fundamental taste	2mM Caffeine Solution(10 ml, Guarana 10% extract powder, Cremar, Seoul, Korea)	4.4
Salty	Sodium chloride solution-related fundamental taste	10% NaCl solution(10ml, Morton iodized salt, Morton Salt, Inc., Chicago, IL, USA)	8.7
Umami	Mono sodium glutamate-related fundamental taste	0.2% MSG in 0.1% NaCl solution (10ml, Miwon, Daesang Co., Seoul, Korea)	5.2
Savory	Savory Savory flavor related to steamed beans	Cooked bean (5g, cooked at 121°C for 30 min).	6.8
After mouthfeel	A lingering mouthfeel beyond 5 seconds after swallowing		
powdery	Mouthfeel related to remaining powder in the mouth after swallowing	10% roasted bean flour (10ml)	9.3
Stinging	stinging mouthfeel associated with sprits	5% red pepper powder solution(10ml)	7.0
Astringent	Astringent mouthfeel associated with alum solution	0.15% Alum solution (10ml, Alum, McCormick & Co., Inc., Baltimore, MD, USA)	6.1
